# Assessment of adherence to iron supplementation among pregnant women in the Yaounde gynaeco-obstetric and paediatric hospital

**DOI:** 10.11604/pamj.2019.34.211.16446

**Published:** 2019-12-26

**Authors:** Florent Ymele Fouelifack, Julius Dohbit Sama, Charles Enome Sone

**Affiliations:** 1 Department of Obstetrics and Gynaecology of Higher Institute of Medical Technology of Nkolondom, Yaoundé, Cameroon; 2 Obstetrics and Gynaecology Unit of Yaoundé Central Hospital, Yaoundé, Cameroon; 3 Department of Obstetrics and Gynaecology of Faculty of Medicine and Biomedical Sciences, University of Yaoundé I, Yaoundé, Cameroon; 4 Obstetrics and Gynaecology Unit of Yaoundé Gynaeco-Obstetric and Paediatric Hospital, Yaoundé, Cameroon; 5 Obstetrics and Gynaecology Unit of Yaoundé Central Hospital, Yaoundé, Cameroon

**Keywords:** Iron, pregnancy, determinants, compliance, Yaoundé, assessment

## Abstract

**Introduction:**

Anemia is a global problem affecting 41.8% of pregnant women. Iron deficiency is the leading cause during pregnancy. Its prevalence among Cameroonian pregnant women was estimated at 50.9% in 2004. Few studies have evaluated women's adherence to iron supplementation prescribed during pregnancy. We carried this study in order to evaluate the rate of adherence to iron supplementation and its determinants during pregnancy.

**Methods:**

The study was cross-sectional descriptive, on postpartum women at the Gynaeco-Obstetric and Pediatric Hospital of Yaoundé during three months. Adherence was measured using the 8-item Morisky Medication Adherence Scale (MMAS-8). The total score was classified as low, moderate and high adherence.

**Results:**

For a total of 304 recruited women, 16.4% were highly compliant, 27.6% moderately compliant, while 56% were low compliant with iron supplementation during pregnancy. The reasons for non-adherence were side effects (19.7%), forgetting (70.1%) and inaccessibility of iron supplements (20.1%). Up to 85 (or 28%) women found it boring to take medication daily. Women with no side effects were about thrice most likely to adhere to the iron supplementation than those with side effects: OR = 3.73 [2.43-5.71]; P = 0.04. Women aged 25 years and above were more likely to be non-compliant to iron supplementation than those youngers: OR = 0.40 [0.31-0.88]; P = 0.02.

**Conclusion:**

To improve adherence to antenatal iron supplementation, it is important to increase communication for behavior change and counseling before or during antenatal care. Forgetting being the main reason for non-adherence, women should keep their iron in a place of easy access.

## Introduction

Anaemia is a worldwide public health problem affecting both developing and developed countries with major consequences for human health. Globally, anaemia affects 1.62 billion people and affects about 41.8% of pregnant women [1]. Seventeen point two million (57.1%) of pregnant women in Africa are anaemic [1], despite the fact that routine iron supplementation during pregnancy has been almost universally recommended to prevent maternal anaemia especially in developing countries over the past three decades [2,3]. The prevalence of anaemia in pregnancy in Cameroonian women was estimated at 50.9% in 2004 [1]. Iron deficiency anaemia during pregnancy increases the risk of maternal mortality, foetal morbidity and mortality, preterm delivery, and low birth weight [4]. Many developing countries are now implementing iron supplementation programs [5,6], but only a few countries have reported significant improvement in anaemia control and prevention [7]. Word Health Organization (WHO) recommends giving all pregnant women a standard dose of 60mg Iron + 400μg folic acid daily throughout pregnancy [8]. Studies conducted in South-East Asia, Latin America and in few African countries have shown that one of the main reasons why these programs have been less effective than anticipated is low compliance of women with taking daily iron supplements. Low compliance has been associated with a number of factors, including: gastrointestinal side effects that can occur with taking iron, inadequate supply of tablets (including limited resources to purchase tablets), inadequate counseling of patients by healthcare providers concerning the utility of tablets and possible transient side-effects, poor utilisation of prenatal health-care services, lack of knowledge and/or patient fears about the tablets and community beliefs, attitudes and practices that affect women´s perception regarding tablet use [9]. In Cameroon, iron supplementation is the main strategy for anaemia control and prevention in pregnancy. Apart from the National Demographic and Health Survey in 2004 that reported on compliance to iron supplementation during pregnancy, no study has been carried out on compliance to iron supplementation in our environment. Our general objective was to find out the compliance rate and factors that influence compliance to iron supplementation among pregnant women, specifically to describe the socio-demographic characteristics of women attending antenatal consultations in the Yaoundé Gynaeco-Obstetric and Paediatric Hospital, to estimate the adherence rate to iron supplementation during pregnancy and to evaluate factors that affect adherence to iron supplementation during pregnancy.

## Methods

We carried out a descriptive cross-sectional study over three months, from the 27^th^ of April 2015 to the 27^th^ of June 2015, in the Gynaecology and Obstetrics Unit of the Yaoundé Gynaeco-Obstetrics, and Paediatric Hospital. Our study population was made of all women in the post-partum period in the Yaoundé Gynaeco-Obstetrics, and Paediatric Hospital within our study period. We included those who freely give their consent. We excluded all those who did not give their consent. The sample size was calculated using the Lorenz formula as follows:

$$N=\frac{{\left(Z_{\alpha }\right)}^{2}XPX[1–P]}{d^{2}}$$

Where: N was the sample size, Z_α_ = the value of Z corresponding to α in a bilateral situation. Taking α = 0.05, the FISHER YATES tables give a Z_α_ value of 1.96; d = degree of precision (allowable error of known prevalence) = 0.05, p = prevalence rate of adherence to medication = 75.2%. Substituting these gives N = 287. But to increase the validity of our study, we consecutively recruited all women in post-partum who gave their consent, that's 304 women. All women who consent were interviewed by the principal investigator. The monthly income of the patients was estimated U.S. dollars left in increments of 90, the local tranches of 50000 Fcfa in currency), assuming that 1$ assumed was 555 francs CFA. Adherence to (or compliance with) a medication regimen was defined as the extent to which patients took medications as prescribed by their health care providers [10]. Adherence was measured by using the 8-item Morisky Medication Adherence Scale (MMAS-8), a structured, self-reported medication adherence measure. Each item on the scale is graded 0 or 1. The sum score (ranging from 0 to 8) was calculated and then trichotomized into low (sum score < 6), medium (sum score 6 or 7) and high (sum score 8) adherence [11]. The data collected was entered into a data base for analysis using the Epi info version 7 and the SPSS (Statistical Package for Social Sciences) version 18 software. Descriptive statistics were used as appropriate. The Chi square or Fisher's exact tests used were appropriate. Binary logistic regression was used to explore determinants of low adherence during pregnancy. A p value < 0.05 was considered statistically significant.

## Results

A total of 304 women who delivered were recruited for the study.

**Socio-demographic characteristics of women in our study:** the Table 1 below shows the socio-demographic characteristics of women in our study population. The mean age of the respondents was 27.9 ± 6.1 (range: 14-42) years. Most of the women were between 21 and 30 years old. Most of the women (54.6%) were married and have attended university. Respectively 39%, 35%, and 26% had incomes below 90 dollars, including between [90 and 180[ and [180-270].

**Table 1:** Socio-demographic characteristics of women

Characteristics	Frequency (n)	Percentage (%)
**Age**		
≤ 20	26	8.6
21 – 30	189	62.2
31 – 40	83	27.3
> 40	06	1.9
Mean age	27.9 +/- 6.1	
**Previous children**		
Yes	192	63.2
No	112	36.8
**Marital status**		
Married	166	54.6
Single	85	28.0
Cohabits	53	17.4
**Level of education**		
University	169	55.6
Secondary school	121	39.8
Primary school	14	4.6
**Income (USD)**		
< 90	39	28.1
[90 – 180]	35	25.1
[180 – 270]	26	18.7
[270 – 360]	11	07.9
[360 – 450]	14	10.1
≥ 450	14	10.1

USD= United States Dollards

**Figure 1:**
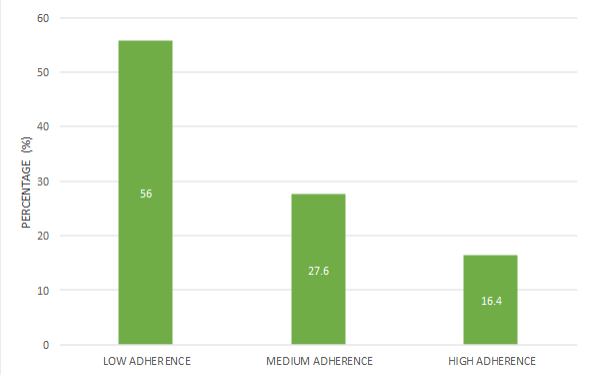
Rate of adherence to iron during pregnancy

**Adherence to iron supplementation during pregnancy:** according to the 8 element Morisky medication adherence scale, the adherence to iron supplementation during pregnancy as illustrated in the Figure 1 below. Only 16.4% of the women where highly complaint while up to 56% showed low adherence to iron supplementation during pregnancy.

**Reasons for non-compliance of iron supplementation among pregnant women:** reasons for non-compliance given by the women in our study are shown in the Table 2. The reasons included side effects from the drug (19.7%), forgetfulness (70.1), non-affordability of iron supplements (20.1%). Up to 85 (28%) women in the study population found it boring taking drugs on a daily basis.

**Table 2:** Reasons for non-compliance of iron folate supplementation among pregnant women

Reason	Frequency	Percentage (%)
Forgetfulness	213	70.1
Side effects	60	19.7
Difficulties buying drugs	61	20.1
Fear that too many tablets would harm her baby	19	6.3
It is boring taking drugs every day	85	28.0

**The most common side effects experience by women:** the most common side effects are shown in the table below (Table 3). The most common side effects were nausea and vomiting though most of the women (80.2%) did not experience any side effects from the drug.

**Table 3:** Side effects of iron supplements reported by the respondents

Side effect	Frequency (n)	Percentage (%)
Bad taste	9	3.0
Bad odour	9	3.0
Nausea and vomiting	32	10.5
Constipation	4	1.3
Asthenia	6	2.0
None	244	80.2
Total	304	100

**Respondents' characteristics versus compliance to iron supplementation in pregnancy:** women with high (16.4%) and medium (27.6%) compliance were grouped together and compared with those with low compliance (56%). Associations between the compliance to iron supplementation in pregnancy and past history of delivery, level of education, income, side effects and maternal age are presented in Table 4. When those with high and medium compliance were grouped together and compared with those with low compliance, we observed that women with no side effects to iron supplementation were about thrice most likely to adhere to the iron supplementation than those with side effects: OR = 3.73 [2.43-5.71]; P = 0.04. Women aged 25 years and above were more likely to be non-compliant to iron supplementation than those with younger ages: OR = 0.40 [0.31-0.88]; P = 0.02.

**Table 4:** Respondents' characteristics versus compliance to iron supplementation in pregnancy, in the binary logistic regression analysis

Characteristics	Adherence to iron		OR [95% C I]	P - value
	Adherent (%)	Non-Adherent (%)		
**Previous deliveries**				0.93
Yes	86 (64.2)	106 (62.4)	1.03 [0.72-1.48]	
No	48 (35.8)	64 (37.6)		
**Level of Education**				0.55
Primary	8 (5.4)	6 (3.9)	1.25 [0.94-2.19] 1.50 [0.51-1.09]	
Secondary	69 (47.3)	52 (32.9)		
University	69 (47.3)	100 (63.2)		
**Income (USD)**				0.02
< 180	124 (92.5)	141 (82.9)	4.01 [0.80-4.55]	
≥ 180	10 (7.5)	29 (17.1)		
**Side effect**				0.04
Yes	20 (14.9)	40 (23.5)	3.73 [2.43-5.71]	
No	114 (85.1)	130 (76.5)		
**Age (years)**				0.02
< 25	37 (27.6)	74 (43.5)	0.40 [0.31-0.88]	
≥ 25	97 (72.4)	96 (56.5)		

## Discussion

As seen from the above results, most of the women who delivered in the Gynaeco-Obstetric and Paediatric Hospital within our study period were relatively young, married, and had attained at least secondary education. Adherence to iron supplementation plays a major role in the prevention and treatment of iron deficiency anaemia particularly among pregnant women (Table 1). We evaluated the rate of compliance to prenatal Iron supplementations and investigated the factors associated with compliance of Iron folate supplementation during pregnancy (Table 2). The World Health Organization recommends giving all pregnant women a standard dose of 60mg iron and 400μg folic acid daily throughout pregnancy [8]. Our results revealed that only 16.4% of the women were highly compliant (had a MMAS-8 of 8) to iron supplementation (Figure 1). Our results are similar to the 16% reported in a study carried out in four Districts in Ethiopia [11]. These results are however lower than the 20.4% high adherence rate reported by Bekele *et al.* in Mecha district, Western Amhara [9]. This difference in results may be due to the fact that, Bekele considered women to be adherent if they took more than 90 iron tablets throughout pregnancy which is difficult for most women to count and tell the number of tablets they consumed throughout pregnancy. On the other hand, 44% of the women in our study population were moderately or highly adherent to iron supplementation during pregnancy. These findings are similar to those reported in the 2004 Cameroon National Demographic and Health survey in which 47% of the women reported taking iron the recommended minimum number of days during their pregnancy [12]. These findings are also similar to those of Zakia *et al*. who recorded an adherence rate of 41.1% in pregnant women attending El-Salam Primary Health Care Centre [13]. The extent of low-adherence in our study population was high (56%). This finding aligns with previous research observing a non-adherence to medication of 40.9% to 55.6% during pregnancy [14,15].

The main reasons why women did not take the iron as directed most of the time were: (1) that they forgot to take the tablets most days (70% of answers); (2) that they grew tired of taking the tablets (28%); (3) difficulties buying drugs (20.1%) and (4) that they experienced gastrointestinal side-effects (19.7% of answers) (Table 3). Our findings are similar to those of Binetou *et al*. who showed that the three main reasons why women stop taking iron supplementation during pregnancy were forgetfulness, gastrointestinal side effects and tiredness from taking tablets every day [15]. Seventy percent of women stopped taking the supplementation due to forgetfulness which is comparable to the 78.8% reported in a study in the Philippines [16]. It is worth noting that 6.3% of the women stopped taking the iron tablets for fear that so many tablets may harm their babies. Though this finding is far below the 58% reported by Bekele *et al.* it is a misconception that should be eradicated from our society [9]. This difference may be due to the fact that, most of the women in our study population were highly educated. Twenty point one percent of the women were not compliant to the iron supplements during pregnancy because they had difficulties purchasing the drugs. This can be explained by the fact that most of the women in our study group earned less than 90 USD (that's 50000 francs CFA) a month. Dispensing the iron supplements free of charge to all the antenatal mothers will also definitely improve compliance to iron supplementation in the population as non-affordability of the drug was a barrier to compliance. This finding is in line with that reported by Binetou *et al*. in Dakar who showed that compliance to iron supplementation was higher in women who received free iron during ANC than those who received prescriptions, and Ugwu who showed that non affordability of the drug was a factor for non-adherence in 23.8% of the women [15,17]. Compliance may be influenced by a number of factors. Factors shown to be associated with iron compliance were the occurrence of side-effects and the participants' age (Table 4). Side-effect is frequently considered as a major obstacle to compliance. According to studies in Saudi Arabia [18], Senegal [15] and India [19], side effect was reported as a reason for missing doses by 40.2%, 27.0% and 25.4% of the pregnant women with low adherence, respectively.

We found a significant association between side effects and adherence to iron supplementation during pregnancy as those with no side effects were about thrice most likely to adhere to the iron supplementation than those with side effects: OR = 3.73 [2.43-5.71]; P = 0.04. Because the gastrointestinal side effects of iron supplements were observed as the major determinant to compliance to iron supplementation in this population, it will be necessary to counsel the women that these side effects are generally transient and not harmful. Appropriate orientation is known to raise psychological tolerance to side-effects. Women aged 25 years and above were more likely to be non-compliant to iron supplementation than those with less than 25 years: OR = 0.40 [0.31-0.88]; P = 0.02. This can be explained by the fact that in our context, women in the early stages of reproductive activity are attentive to the advice. But as the age goes on, the number of pregnancies increases and the routine leads to negligence, and therefore a decrease in adherence to iron intake during pregnancy. In contrary, finding with a study in India revealed that elderly and middle aged women were slightly more compliant than younger women, and the study in Western Ahmara which showed a higher compliance rate in elderly women [9,20].

**Strength of the study:** an important strength of this study is the use of a validated questionnaire for self-reporting of medication adherence. This study is the first attempt in our environment to understand factors influencing the utilization of prenatal iron supplement. Also, the selection of women who were in the immediate post-partum period will make the results more reliable since the events of the indexed pregnancy are still fresh in their minds. Furthermore, since they have already put to birth and most without complications, they will give more genuine responses than those who are still pregnant just to give a good impression to the investigator.

**Limits of the study:** the limit of this study is that the self-reporting method we used is less objective than the electronic monitoring which is not available in our environment.

## Conclusion

Occurrence of side effects and maternal age are major factors that influence compliance to prenatal iron supplementation in our setting. It is important to intensify communication for behavioral change and counseling before or during prenatal consultations. This could promote the compliance of pregnant women with the preventive treatment of anemia and its complications. Women should be counseled thoroughly on the side effects as well as the importance of iron during pregnancy so as to improve adherence. They should also be advised to keep their iron in a place where they can easily see them since forgetfulness is the major reason for not taking the tablets.

### What is known about this topic

- Iron deficiency is the leading cause of anemia during pregnancy;
- Its prevalence among Cameroonian pregnant women was estimated at 50.9% in 2004;
- Iron supplementation is the main strategy for anemia control and prevention in pregnancy.

### What this study adds

- Women with no side effects to iron supplementation were about thrice most likely to adhere to the iron supplementation than those with side effects;
- Women aged 25 years and above were more likely to be non-compliant to iron supplementation than those with younger ages (less than 25 years).

## Competing interests

The authors declare no competing interests.
